# Sequential embryo transfer combined with intrauterine perfusion improved pregnancy outcomes in patients with recurrent implantation failure

**DOI:** 10.1186/s12905-024-02966-8

**Published:** 2024-02-16

**Authors:** Wenda Zou, Dan Liu, Juan Peng, Zhijing Tang, Yukun Li, Juan Zhang, Ziwei Liu

**Affiliations:** 1 Department of Reproductive Medicine Center, Zhuzhou Hospital Affiliated to Xiangya School of Medicine, Central South University, South Changjiang Road, Tianyuan District, ZhuZhou, China; 2 Department of Urology, Zhuzhou Hospital Affiliated to Xiangya School of Medicine, Central South University, South Changjiang Road, Tianyuan District, ZhuZhou, 412007 China

**Keywords:** *In vitro* fertilization-embryo transfer, Sequential embryo transfer, Intrauterine perfusion, Frozen–thawed cycle, Recurrent implantation failure, Pregnancy outcomes

## Abstract

**Objective:**

To compare the application of sequential embryo transfer, cleavage embryo transfer, and blastocyst transfer combined with intrauterine perfusion in frozen–thawed embryo transfer cycles in patients with recurrent implantation failure to provide a reference for reproductive clinicians.

**Methods:**

The 166 patients who underwent frozen–thawed embryo transfer due to recurrent implantation failure in the reproductive center from January 2021 to March 2022 were retrospectively analyzed. According to the different embryos transferred, they were divided into cleavage embryo transfer groups (72 cases in Group A), blastocyst transfer group (29 cases in Group B), and sequential transfer group (65 cases in Group C). All three groups were treated with intrauterine perfusion 5 days before embryo transfer. The general data and clinical pregnancy outcome indicators, such as embryo implantation rate, clinical pregnancy rate, ongoing pregnancy rate, live birth rate, twin rate, were compared among the three groups.

**Results:**

The embryo implantation rate (53.1%), clinical pregnancy rate (76.9%), ongoing pregnancy rate (67.7%) and live birth rate(66.15%) in the sequential transfer group were significantly higher than those in the other two groups (*P* < 0.05), and the ectopic pregnancy rate was lower in the sequential transfer group.

**Conclusion:**

Sequential transfer combined with intrauterine perfusion partially improves clinical pregnancy outcomes and reduces the risk of ectopic pregnancy in frozen embryo cycle transfers in patients with recurrent implantation failure, which may be a favourable transfer reference strategy for patients with recurrent implantation failure.

## Introduction

With the continued refinement of superovulation stimulation protocols in vitro fertilization- embryo transfer (IVF-ET) and improvements in embryo culture systems for assisted reproduction, the quantity and quality of embryos have been further improved. However, embryo implantation rate growth has been limited, especially because recurrent implantation failures(RIF) reduced IVF pregnancy rate, which is a difficulty faced by reproductive clinicians [1]. The currently accepted definition of RIF is: three or more transfers with a cumulative total of at least four good quality embryos at the cleavage stage without a clinical pregnancy. To date, the molecular mechanism of embryo implantation has not been elucidated, and the causes are complex and multifaceted, including the following: maternal factors, including psychological factors, abnormalities in the anatomy of the reproductive system, abnormalities in the development and function of the uterine lining, frequent uterine contractions, a tendency to thrombosis, and abnormalities in the immune function of the maternal-fetal interface; embryonic factors, including defects in the genetic material of embryos, poor hatching and developmental potential; and other relevant factors (e.g., the presence of a defective embryo). Embryonic factors include defects in embryonic genetic material, poor embryonic hatching and developmental potential, and other related factors (patient management, clinical management and quality control, laboratory management and quality control), etc. RIF can occur as a result of a single factor, or as a result of a multifactorial combination of factors, and in some cases the cause is unknown.

In order to improve the pregnancy outcome of patients with RIF, scholars in the field of reproduction have been working hard in recent years, and some good progress has been made, such as the use of pre-implantation aneuploidy testing (PGT-A), endometrial tolerance gene expression profiling, and endometrial micro-ecology testing to rule out the possibility that the embryo is caused by aneuploidy, or that the embryo has been damaged by aneuploidy [2]. Failure of implantation due to asynchrony between endometrial implantation window and embryo development and changes in the microenvironment of the uterine cavity caused by endometrial flora dysbiosis [3, 4]. These tests and clinical tools can, to a certain extent, solve the problems of some RIF patients and improve pregnancy outcomes. However, due to the length of the test, price, qualification and damage to the endometrium in sampling, neither PGT-A, endometrial tolerance test nor endometrial microecology can be used for fresh cycle transplantation in the current cycle of the test, and need to be pushed back to the time of transplantation, which prolongs the length of time for patients to achieve clinical pregnancy. Approximately 50–60% of infertility couples aborted IVF-ET treatment after repeated failed attempts due to it being a time-consuming and expensive treatment [5]. Other patients anxiously seek explanations for failure and strategies for improvement. Therefore, it is important to find a non-invasive, affordable method that is not too demanding on the qualifications and equipment of the center, and scholars have been trying different methods, of which sequential transplantation is one of the more promising ones.

To increase embryo implantation rates and obtain better pregnancy outcomes, sequential transfer has been used in many reproductive centers. Sequential transfer, is a combination of cleavage embryo transfer and blastocyst transfer, which can improve endometrial receptivity by maximizing the “window of implantation“ [6]. Crosstalk with the endometrium can be initiated by transferring the first embryo into the uterine cavity, thereby forwarding implantation of the second embryo [7].

However, there remains insufficient evidence to substantiate the advantages of sequential transfer. Intrauterine perfusion was conducive to achieving high local drug concentrations, improving the microenvironment of the endometrium and facilitating embryo implantation. Dexamethasone is an immunosuppressive and anti-inflammatory agent that is widely used clinically in autoimmune diseases and transplant rejection. Granulocyte colony-stimulating factor (G-CSF) is a glycoprotein secreted by endothelial cells, macrophages and related immune cells, and plays an important role in the proliferation of a variety of cells [8]. However, these two drugs have been less studied in the reproductive field for their local action on the endometrium. This study provides evidence for the transfer strategy by examining the clinical significance of sequential transfer combined with intrauterine infusion above two drugs in a frozen–thawed cycle.

## Materials and methods

### Study subjects

A retrospective analysis of cases from January 2021 to March 2022 of patients who came to our reproductive center for frozen embryo transfer due to previous IVF-ET failure.

Inclusion criteria: (1) age: 20–40 years old; (2) failure to achieve a clinical pregnancy after transfer of at least four good-quality embryos in a minimum of three fresh or frozen cycles [9]; (3) available Day 3 cleavage stage embryos and Day 5 blastocyst embryos. The study was conducted in accordance with the ethical standards of the Declaration of Helsinki.

The exclusion criteria were as follows: (1) uterine organic lesions, such as unicornuate uterus, residual horn uterus, septate uterus, saddle-shaped uterus, adenomyosis, uterine submucosal fibroids, and intrauterine adhesions; (2) contraindications to assisted reproductive technology and pregnancy; (3) abnormal karyotype in the peripheral blood of either spouse; and (4) severe hydrosalpinx and no treatment [10].

### Grouping

They were divided into cleavage stage embryo transfer group (group A, 72 cases), blastocyst transfer group (group B, 29 cases), and sequential transfer group (group C, 65 cases). For the cleavage embryo transfer group, two cleavage embryos were transferred on day 3 of endometrial transformation; for the blastocyst transfer group, two D5 blastocysts were transferred on day 5 of endometrial transformation; for the sequential transfer group, one D3 cleavage embryo was transferred first, followed by one D5 blastocyst on day 5 of endometrial transformation.

### Controlled ovulation induction and embryo selection

Controlled ovulation promotion was performed using long-term regimens (long-acting long-term regimen, follicular long-term regimen) and antagonist regimens. Triggered with human chorionic gonadotropin (HCG) 5000–10,000 U and oocytes retrieved after 36 h. Insemination was performed by in vitro fertilization or intracytoplasmic sperm injection (IVF/ICSI), followed by the evaluation of embryos at the cleavage stage on day 3. Definition of quality embryos: uniform and fine cytoplasmic granules; regular morphology; normal cleavage rate; homogeneous blastocysts; and less than 5% fragmentation. Embryos at the blastocyst stage on day 5 were graded according to the Gardner scoring system [11]. The development of blastocysts is divided into six periods based on the size of the blastocyst cavity and whether or not they hatch. Stage 1: early chambered blastocysts, with the blastocyst cavity being less than 1/2 of the total volume of the embryo; stage 2: the volume of the blastocyst cavity is greater than or equal to 1/2 of the total volume of the embryo; stage 3: completely dilated blastocysts, where the cavity completely occupies the total volume of the embryo; stage 4: dilated blastocysts, where the cavity is completely full of embryo and the total volume of the embryo becomes larger and the zona pellucida becomes thinner; stage 5: hatching blastocyst, part of the blastocyst escapes from the zona; stage 6: hatching blastocyst, all of the blastocyst escapes from the zona; Blastocysts at stages 3 to 6 are also graded for the quality of their inner cell mass and trophoblast cells. Grading of the inner cell mass: grade A, high number of cells, tightly packed; grade B, low number of cells, loosely packed; grade C, low number of cells. Grading of trophoblast cells: grade A, the epithelial cell layer consists of a large number of cells with a dense structure; grade B, the epithelial cell layer consists of a small number of cells with a loose structure; grade C, the epithelial cell layer consists of a sparse number of cells [11]. Blastocysts with a grade ≥ 3BB were considered high-quality blastocysts [11]. In the cleavage stage embryo transfer group, 2 day 3 cleavage stage embryos were transferred. In the blastocyst-stage embryo transfer group, 2 blastocyst-stage embryos were transferred on day 5. In the sequential transfer group, one cleavage-stage embryo was first transferred on day 3 and then one blastocyst-stage embryo was transferred on day 5.

### Endometrial preparation protocol and embryo transfer

Regarding the preparation of the endometrium, a hormone replacement therapy protocol was used. Estrogen addition was used to increase the thickness of the endometrium. Estradiol valerate (Progynova, Bayer Healthcare Guangzhou, 1 mg*21 tablets) was administered at a dose of 3 mg/dose twice daily and endometrial morphology and thickness were reviewed by transvaginal ultrasonography after 7–10 days. If the endometrial thickness was less than 7 mm, the dose of estradiol/norethindrone was increased (Femoston, Abbott biologicals B.V., 2/10 mg); meanwhile, we considered whether it would be appropriate to increase the dose of estradiol valerate and extend the duration of use accordingly until the endometrial thickness reached 8–13 mm. The endometrial modification was performed after the endometrium reached the standard: progesterone soft gels (Utrogestan, cyndea Pharma, s.l., 0.1 g * 30 tablets), 0.2 g twice daily; and desogestrel tablets (Dating, Abbott biologicals B.V., 10 mg * 20 tablets), twice daily, each 20 mg orally. Each transfer group was infused with granulocyte colony-stimulating factor (Ruibai, Qilu Pharmaceutical Co., Ltd., 100 mg) and dexamethasone sodium phosphate injection (DexSPI, Cinnabar Pharmaceutical Co., Ltd., 5 mg) in the uterine cavity and infused with 1 ml of fluid 5 days before embryo transfer. The method of instillation was as follows: the patient was instructed to empty the bladder, take the lithotomy position, routinely disinfect the vulva and vagina, use a 1 ml syringe to connect with a Cook’s disposable insemination tube, to aspirate 0. 9 ml of the medication, which was slowly injected into the uterine cavity. After endometrial transformation, embryos were transferred to the three groups according to the above transfer strategy.

### Pregnancy outcomes and surveillance indicators

On the 14th day after embryo transfer, biochemical pregnancy was judged based on blood test. On the 28th day after embryo transfer, the gestational sac was found by ultrasound and judged as clinical pregnancy. Clinical pregnancy rate = the number of clinical pregnancy cycles/number of transfer cycles × 100%, embryo implantation rate = the number of embryos implanted/total number of embryos transferred × 100%; ongoing pregnancy rate = the number of cycles with more than 20 weeks of persistent pregnancy/number of transfer cycles × 100%, twin pregnancy rate = the number of twin pregnancy cycles/number of pregnancy cycles × 100%; ectopic pregnancy rate = the number of ectopic pregnancy cycles/number of pregnancy cycles × 100%^3^.

### Statistical analysis

All statistical data analyses and figures were carried out using SPSS 26.0 and GraphPad Prism 8.0. Based on our small sample size, we used Shapiro-Wilk test for conformity to normal distribution, age was conformed, which measured data were expressed as mean ± standard deviation (x ± s), other baseline information was not conformed, which was expressed as median (25th percentile, 75th percentile) [M (P_25_, P_75_)],and Kruskal-Wallis H Test was used to compare mean values between groups. Enumeration data were expressed as ratios (%), and Fisher exact test was used to compare ratios values between groups. All statistical results with p value < 0.05 were considered statistically significant.

## Results

### Comparison of general data

In total, 166 cases were included in this study. There was no significant difference in age, years of infertility, cause of infertility, Previous failed cycles, Good-quality embryos on day 3, No. of eggs fertilized, BMI index, anti-Müllerian hormone (AMH), retrieved oocytes, or antral follicle count (AFC) among the three groups (*p* > 0.05) (Table 1).

**Table 1:** Comparison of general data

Group	A	B	C
Age(years)	32.54 ± 3.43	30.90 ± 3.09	31.43 ± 4.05
Infertility(years)	5(4,8)	5(3,7)	4(2,7)
BMI (kg/m)	22.43(20.59,25.03)	21.09(19.5,23.48)	21.87(20.4,24.03)
Retrieved oocytes (n)	15(12,19)	17(15,22)	15(11.5,23)
AMH(ng/ml)	3.19(2.39,5.51)	4.4(3.19,6,73)	3.8(2.31,6.35)
AFC (n)	16(9,19)	16(11.5,22)	15(11.5,23)
Cause of infertility(%)			
Male	41.67 (30/72)	24.14(7/29)	32.31 (21/65)
Female	44.45(32/72)	44.83(13/29)	50.77(33/65)
Unkown	13.89(10/72)	31.03(9/29	16.92(11/65)
Previous failed cycles	3.85 ± 1.53	3.75 ± 0.84	3.63 ± 0.89
Good-quality embryos on day 3	4.5 ± 1.9	4.7 ± 1.8	4.8 ± 1.7
No. of eggs fertilized	12(11,16)	14(11,19)	13(10,20)

### Comparison of pregnancy outcomes

**Fig. 1:**
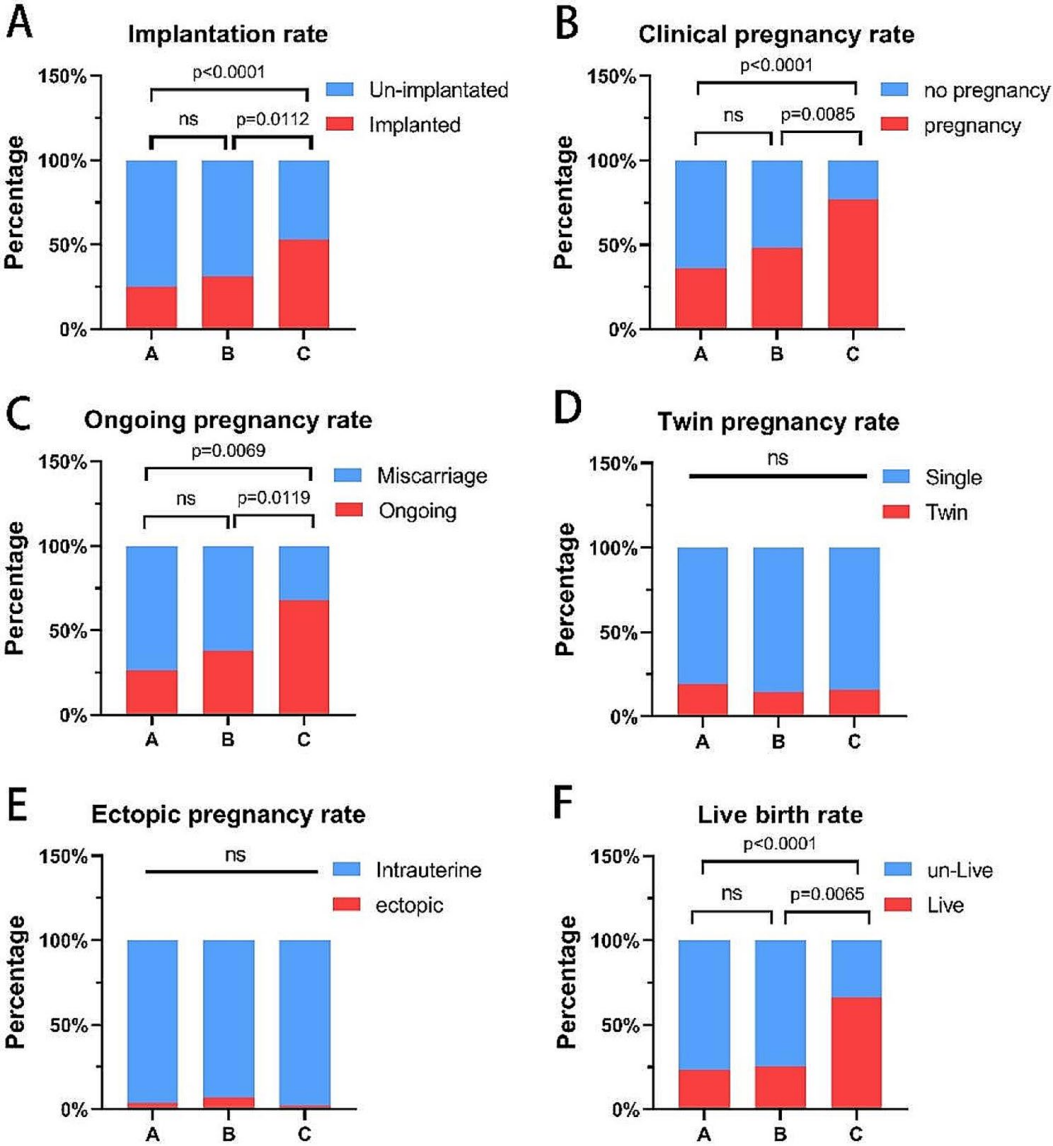
Group C (sequential transfer group) were significantly higher than Group A (cleavage stage transfer group) and B ( blastocyst transfer group) in terms of implantation rate, clinical pregnancy rate, ongoing pregnancy rate and live birth rate( *P* < 0.05), No significant difference was found between Group A and B ( *P*>0.05); D,E: There was no significant difference among the three groups in terms of ectopic pregnancy rate and twin pregnancy rate( *P* > 0.05). ns: no significant difference

Upon comparison of clinical pregnancy outcomes among the three groups, the embryo implantation rate, clinical pregnancy rate, ongoing pregnancy rate and live birth rate of the sequential transfer group (Group C) were significantly higher than those of the other two groups (*P* < 0.05). There was no significant difference among the three groups in ectopic pregnancy rate (*P* > 0.05), but the ectopic pregnancy rate in the sequential embryo transfer group (Group C) was lower than that in the other two groups, and the twin pregnancy rate of the blastocyst group (Group B) was lower than that of the other two groups, which has no significant difference. There was no significant difference between Group A and Group B in the implantation rate, clinical pregnancy rate, ongoing pregnancy rate, early miscarriage rate and live birth rate (Table 2; Fig. 1).

**Table 2:** Comparison of clinical pregnancy outcomes

Group	A	B	C
Number of cases (n)	72	29	65
Implantation rate (%)	23.6(34/144)	25.8(15/58)	53.1(69/130)
Clinical pregnancy rate (%)	36.1(26/72)	48.3(14/29)	76.9(45/65)
Ongoing pregnancy rate (%)	26.4(19/72)	37.9(11/29)	67.7(44/65)
Twin pregnancy rate (%)	19.2(5/26)	14.3(2/14)	15.6(7/45)
Ectopic pregnancy rate (%)	3.8(1/26)	7.1(1/14)	2.2(1/45)
Live birth rate (%)	23.6(17/72)	34.5(10/29)	66.2(43/65)
Early miscarriage rate	7.7(2/26)	7.1(1/14)	6.7(3/45)

## Discussion

Two important conditions should be met during embryo implantation: a good quality embryo with developmental potential and endometrium that allows embryo positioning, adhesion, and implantation [12]. The instability of any of these factors can affect embryo implantation. Therefore, improving embryo quality and endometrial receptivity are key factors in reducing embryo implantation failure [5, 7]. During the early stages of oocyte division, embryonic development is controlled by maternal genes and the involvement of the embryonic genome must be activated after cell cleavage. Selection of high-quality embryos by morphology alone may not accurately predict the developmental potential of the embryo [13, 14]. Therefore, prolonging the in vitro culture time is a reliable way to eliminate embryos with low developmental potential where blastocyst transfer can result in better embryo implantation rates and pregnancy rates [15, 16]. In this study, we compared cleaved embryos with blastocysts, noting higher implantation and pregnancy rates and lower multiple pregnancy rates in the latter. Several studies have also confirmed this [17–19].

Blastocyst culture results indicate the quality and developmental potential of the embryos, which is a valid indicator of successful conception. However, blastocyst transfer requires a certain number of high-quality embryos available for culture and is not suitable for patients with decreased ovarian reserve [5]. A higher fertilization rate and better embryo development have been shown in patients with oocytes with normal perivitelline space [20]. The patients in the Scantamburlo VM,et al. study with decreased ovarian reserve showed a stronger association with the occurrence of changes in the perivitelline space, which may result in worse reproductive outcomes [21]. Therefore, patients with decreased ovarian reserve, there are poorer and fewer eggs, then resulting in poorer and fewer blastocysts.

The sequential transfer combines the advantages of cleaved embryos and blastocyst transfer, providing patients with the opportunity to transfer blastocysts, reducing the risk of embryo-free transfers, and also taking advantage of the high implantation rate of blastocysts to improve pregnancy rates [5]. Some researchers have reported that two-thirds of IVF embryo transfer failures are due to lack of endometrial receptivity [22]. The endometrium becomes receptive to embryo implantation 6–8 days after ovulation and remains receptive for 2–4 days. In at least 25% of patients with RIF, a different time window for implantation has been demonstrated based on transcriptomic alterations of the endometrium in the mid-luteal phase [23]. This study revealed that the sequential transfer group had higher embryo implantation rates, clinical pregnancy rates, ongoing pregnancy rates and live birth rates compared to the blastocyst transfer group, which is similar to the results of sever studies [24–27]. Thus, variability in the endometrial maturation process and sequential transfers pinpointing the window of implantation, which increases the window of tolerance, have been cited in the literature as the main factors for increased rates [28]. In addition, sequential transfer provides a good model of embryo transfer with simultaneous in vivo and in vitro culture, which may be one of the factors contributing to the higher clinical pregnancy rates.

The mechanism for above results has been explained by the following: performing a sequential transfer in the same cycle maximizes the chances of coordination between embryo recognition and endometrial receptivity and improves implantation rates [5]. Sequential transfer may increase the chance of embryo implantation due to mechanical stimulation of the endometrium by the insertion of the transfer catheter during the initial transfer, resulting in aseptic endometrial damage that may induce endometrial expression of relevant factors. The heterogeneity of the endometrial tissue structure determines that the time to reach optimal receptivity also varies from tissue to tissue [25].With regard to the aseptic damage to the endothelium caused by the transplanted catheter, which may induce endothelium-associated expression, we are not able to perform embryo-free nulliparous transfers for the time being with the fully informed consent of the patients, who have already experienced repeated implantation failures and are reluctant to reduce the number of embryos transferred, maybe we can perform such groupings in future animal experiments in order to better demonstrate the advantages of sequential transplantation.

When embryo quality and embryo culture environment are matched, embryo consistency and endometrial receptivity are key to improving pregnancy rates. Intrauterine perfusion drugs have been proven to be effective in promoting mucosal recovery and cell division and proliferation [29].

The functions of granulocyte colony-stimulating factor (G-CSF) are mainly to prevent wound infection, activate vascular endothelial growth factor, promote the growth of new blood vessels, promote the proliferation and differentiation of trophoblast cells, and stimulate the expression of related factors; accelerate the maturation and transformation of keratinocytes, and promote the proliferation, recovery, and differentiation of wound tissue [30–32]. G-CSF ameliorates endometrial thickness and optimizes embryo accommodation through the induction of inflammatory factors and the proliferation of tissue for repair. In 2009, Scarpellini et al. used G-CSF for the first time in 86 women 217 with unexplained recurrent miscarriage and showed that 82% of women treated with G-CSF delivered a healthy 218 newborn compared to 48% of controls; in addition, in their study, G-CSF was 219 completely safe and not associated with any adverse maternal or neonatal outcomes [33]. Some studies have applied it in intrauterine perfusion to improve the pregnancy and implantation rates in infertile patients [34–36].

As an immune response and anti-inflammatory agent, dexamethasone is primarily used clinically for the treatment of autoimmune diseases and against transplant rejection [37]. One study applied intrauterine infusion of dexamethasone in patients to improve their pregnancy outcome since dexamethasone locally regulates the balance of helper T lymphocytes in the endometrium, promotes the secretion of related factors, reduces the activity of natural killer cells, and inhibits maternal-fetal immune rejection, thus maintaining the balance of maternal and fetal immunity [38]. In their analysis of 16 RCTs on the use of glucocorticoids in assisted reproduction Boomsma et al. found that no studies reported the incidence of infections or fetal abnormalities [39]. These drugs are widely used in the fields of obstetrics and gynecology and have no teratogenic effect [40].

In this study, we combined dexamethasone and G-CSF to improve the endometrial microenvironment, maintain maternal-fetal immune balance, and promote embryo implantation, which improved the pregnancy rate in patients with recurrent implantation failure. Further, we also found that the ectopic pregnancy rate in the sequential transfer group was lower than that in the blastocyst and cleavage stage embryo groups, suggesting that sequential transfer has a role in the reselection of the embryo implantation site and reduces the risk of ectopic pregnancy in patients. In addition, we found a high rate of twin pregnancies in the three groups, considering that this may be related to the number of embryos transferred and the small sample size. Stamenov et al. suggested that the reason might be that the specific window of implantation of patients with multiple pregnancies in the sequential transfer group coincided with the period when both day 3 and day 5 embryos were ready for implantation [25]. But sequential transfer did not significantly increase the incidence of twin pregnancies in our study. These findings are consistent with the results of a 2021 systematic review [6]. After recurrent implantation failure, most patients wanted to transfer two embryos to increase clinical pregnancy rates. The above findings prompt us to rethink the benefits of double embryo transfer in patients with repeated implantation failure [18].

However, this study had some limitations that should be taken into account when interpreting the results; due to the nature of the study, it was not blinded and no placebo was used, which could potentially influence the results of the study. Intrauterine instillation is an invasive procedure, which increases the risk and cost of infection, and its impact itself increases the study bias, but there were no cases of infection in the enrolled patients, and we will also add a blank control group as appropriate to obtain more convincing data in future studies. Despite above mentioned literatures, further prospective experimental analyses are needed to demonstrate the efficacy and safety of these two drugs; a randomized clinical trial is essential for further validation, providing a stronger level of evidence.

## Conclusion

Sequential transfer combined with intrauterine perfusion partially improves clinical pregnancy outcomes and reduces the risk of ectopic pregnancy in frozen embryo cycle transfers in patients with recurrent implantation failure, which may be a favorable transfer reference strategy for patients with recurrent implantation failure.

## Data Availability

The datasets used and analyzed during the current study are available from the corresponding author on reasonable request.
